# D-VRD induction and autologous transplant in patients ≥70 years

**DOI:** 10.1038/s41408-026-01522-x

**Published:** 2026-06-05

**Authors:** Oren Pasvolsky, Curtis Marcoux, Denái R. Milton, Mark R. Tanner, Muhammad Bilal Islam, Qaiser Bashir, Samer Srour, Neeraj Saini, Paul Lin, Jeremy Ramdial, Yago Nieto, Guilin Tang, Yosra Aljawai, Asad A. Haider, Hans C. Lee, Krina K. Patel, Partow Kebriaei, Sheeba K. Thomas, Robert Z. Orlowski, Richard E. Champlin, Elizabeth J. Shpall, Muzaffar H. Qazilbash

**Affiliations:** 1 Department of Lymphoma and Myeloma, The University of Texas MD Anderson Cancer Center, Houston, TX USA; 2 Division of Hematology, Dalhousie University, Halifax, NS Canada; 3 Department of Biostatistics, The University of Texas MD Anderson Cancer Center, Houston, TX USA; 4 Department of Stem Cell Transplantation and Cellular Therapy, The University of Texas MD Anderson Cancer Center, Houston, TX USA; 5 Department of Hematopathology, The University of Texas MD Anderson Cancer Center, Houston, TX USA; 6 Department of Experimental Therapeutics, The University of Texas MD Anderson Cancer Center, Houston, TX USA

**Keywords:** Myeloma, Medical research

Dear Editor,

The addition of daratumumab to triplet induction regimens has significantly improved depth of response and progression-free survival (PFS) in newly diagnosed multiple myeloma (MM), as demonstrated in the GRIFFIN and PERSEUS trials, both of which evaluated daratumumab, bortezomib, lenalidomide, and dexamethasone (D-VRD) induction followed by autologous hematopoietic cell transplantation (autoHCT) [1, 2]. However, both trials excluded patients over age 70, limiting the generalizability of these findings to older adults, a population in which MM is commonly diagnosed [3]. Although limited real-world data suggest a benefit of D-VRD induction [4] over triplets, evidence in older patients is lacking.

To address this gap, we conducted the largest retrospective study to date to evaluate the outcomes of newly diagnosed MM patients aged ≥70 years, who were treated with D-VRD induction followed by autoHCT. We hypothesized that the safety and efficacy of this approach would be comparable between older and younger patients.

We included consecutive newly diagnosed MM patients, who received D-VRD induction followed by upfront autoHCT between 2021 and 2024 at The University of Texas MD Anderson Cancer Center. Patients were stratified by age at transplant: ≥70 years (older) vs <70 years (younger). Primary outcomes were PFS and overall survival (OS). Stabilized inverse probability weighting (IPW) adjusted for potential confounders including Revised International Staging System (R-ISS) stage, hematopoietic cell transplantation–specific comorbidity index (HCT-CI), cytogenetics, conditioning regimen, and maintenance therapy was used to account for baseline differences between age groups in the Cox proportional hazards regression models for PFS and OS. Measurable residual disease (MRD) was assessed by eight-color next-generation flow cytometry on bone marrow aspirates with 0.001% sensitivity at pre-autoHCT, approximately day +100 post-autoHCT, and subsequently at the discretion of treating physicians. Neutrophil engraftment was defined as the first of three consecutive days with an absolute neutrophil count ≥500/µL following post-transplant nadir, and platelet engraftment as the first of three consecutive days with unsupported platelet count ≥20 × 10⁹/L.

A total of 125 patients were included: 27 (22%) in the older group and 98 (78%) in the younger group. Median age was 74 (range 70–78) vs 59 (range 40–69) years, respectively. Baseline patient and disease characteristics were generally similar, although lenalidomide dosing differed: 10 (37%) older patients received ≥20 mg as the most common dosage during induction vs 73 (74%) patients in the younger group. The maximum lenalidomide dose was ≥20 mg in 14 (52%) older vs 83 (85%) younger patients, respectively. Conditioning with melphalan alone was more common in older patients (93% vs 61%, p = 0.007), while 30% of younger patients received busulfan–melphalan vs 7% in the older group. Among patients aged ≥70 years who received single-agent melphalan conditioning, 18 (72%) received melphalan 140 mg/m², while 7 (28%) received melphalan 200 mg/m². Rates of high-risk cytogenetic abnormalities (56% vs 47%, p = 0.52) and R2-ISS stage III/IV disease (59% vs 43%, p = 0.37) were similar. Post-transplant maintenance therapy was administered in 96% vs 97% (p = 1.00), most commonly lenalidomide alone (77% vs 85%, p = 0.37). Baseline patient and disease characteristics are shown in Table 1.

**Table 1:** Baseline patient, disease, and treatment characteristics.

Characteristic	All patients (N = 125)	< 70 years (N = 98)	≥ 70 years (N = 27)	p value^a^
**Gender, n (%)**				
Female	47 (38)	36 (37)	11 (41)	0.82
Male	78 (62)	62 (63)	16 (59)	
**Age, years, median (range)**	62 (40–78)	59 (40–69)	74 (70–78)	<0.001^b^
**Race, n (%)**				
Black	20 (16)	15 (15)	5 (19)	0.45
Non-Black	103 (82)	82 (84)	21 (78)	
Unknown	2 (2)	1 (1)	1 (4)	
**R-ISS, n (%)**				
I	37 (30)	30 (31)	7 (26)	0.17
II	56 (45)	46 (47)	10 (37)	
III	19 (15)	11 (11)	8 (30)	
Unknown	13 (10)	11 (11)	2 (7)	
**R2-ISS, n (%)**				
I/II	53 (42)	44 (45)	9 (33)	0.37
III/IV	58 (46)	42 (43)	16 (59)	
Unknown	14 (11)	12 (12)	2 (7)	
**Cytogenetic risk**^**c**^**, n (%)**				
Standard	64 (51)	52 (53)	12 (44)	0.52
High	61 (49)	46 (47)	15 (56)	
**ECOG performance status**				
0	40 (32)	34 (35)	6 (22)	0.26
1	57 (46)	45 (46)	12 (44)	
2	2 (2)	1 (1)	1 (4)	
Unknown	26 (21)	18 (18)	8 (30)	
**HCT-CI**				
Median (range)	3 (0–8)	3 (0–8)	2 (0–8)	0.75^b^
**HCT-CI, n (%)**				
≤3	83 (66)	65 (66)	18 (67)	1.00
>3	42 (34)	33 (34)	9 (33)	
**Number of induction cycles, n (%)**				
<4	13 (10)	11 (11)	2 (7)	0.73
≥4	112 (90)	87 (89)	25 (93)	
**Duration of induction, n (%)**				
<126 days	62 (50)	51 (52)	11 (41)	0.39
≥126 days	63 (50)	47 (48)	16 (59)	
**Most common lenalidomide dose, n (%)**				
<20 mg	42 (34)	25 (26)	17 (63)	<0.001
≥20 mg	83 (66)	73 (74)	10 (37)	
**Maximum lenalidomide dose, n (%)**				
<20 mg	28 (22)	15 (15)	13 (48)	0.001
≥20 mg	97 (78)	83 (85)	14 (52)	
**Conditioning regimen, n (%)**				
Busulfan + melphalan	31 (25)	29 (30)	2 (7)	0.007
Melphalan	85 (68)	60 (61)	25 (93)	
140 mg/m2	24 (28)	6 (10)	18 (72)	
200 mg/m2	61 (72)	54 (90)	7 (28)	
Melphalan hydrochloride	9 (7)	9 (9)	0	
**Maintenance use, n (%)**				
No	4 (3)	3 (3)	1 (4)	1.00
Yes	121 (97)	95 (97)	26 (96)	
**Maintenance treatment, n (%)**				
Lenalidomide	101 (83)	81 (85)	20 (77)	0.44
Lenalidomide-based	10 (8)	7 (7)	3 (12)	
Other	10 (8)	7 (7)	3 (12)	

*ECOG* Eastern Cooperative Oncology Group, *HCT-CI* Hematopoietic Cell Transplantation Comorbidity Index, *MRD* measurable residual disease, *n/N* number, *R-ISS* Revised International Staging System, *R2-ISS* Second Revision of International Staging System.

^a^Fisher’s exact test or its generalizations.

^b^Wilcoxon rank sum test.

^c^t(4;14), t(14;16), del(17p), and 1q21 gain or amplification.

There were no non-relapse mortality (NRM) events in either group. Four patients developed second primary malignancies, all of whom were in the younger cohort. Pre-transplant response rates were similar between groups: ≥ very good partial response (VGPR) was achieved in 22 (81%) older vs 76 (78%) younger patients (p = 0.79), and ≥ complete response (CR) in 8 (30%) vs 24 (24%) (p = 0.62). Post-transplant, ≥VGPR was observed in 26 (96%) older vs 92 (94%) younger patients (p = 1.00), and ≥CR in 17 (63%) vs 48 (49%) (p = 0.28). At best post-transplant response, all older patients (27/27, 100%) and nearly all younger patients (97/98, 99%) achieved ≥VGPR, with ≥CR in 21 (78%) older and 75 (77%) younger patients, respectively (p = 1.00) (Fig. 1).

**Fig. 1:**
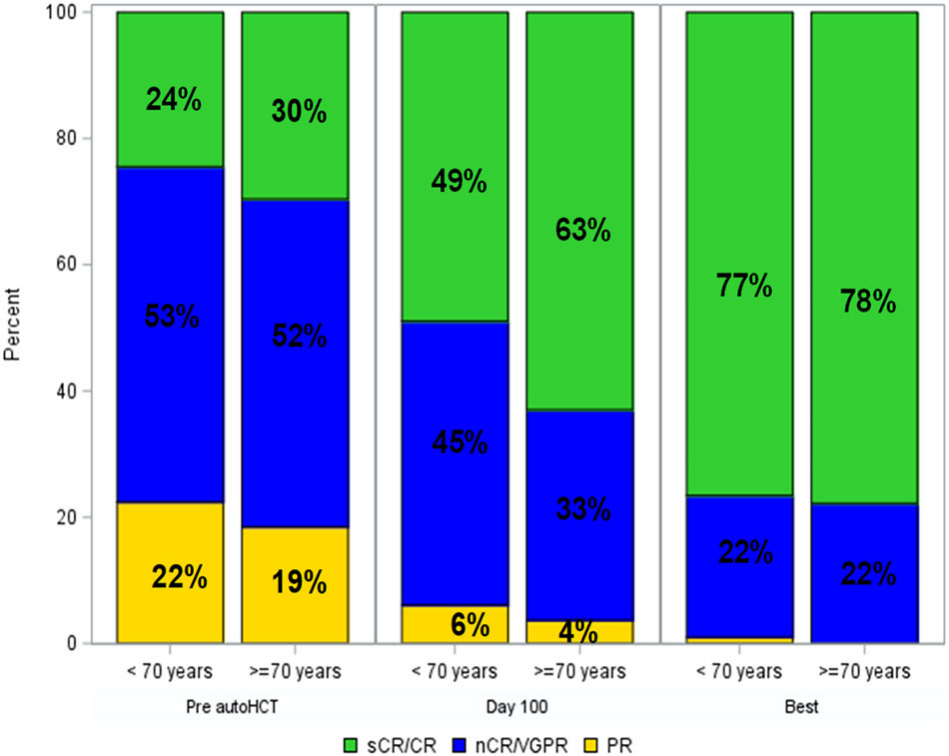
Pre- and post-transplant hematological responses by age group.

Overall, 42% (11/26) of evaluable older patients and 49% (47/96) of evaluable younger patients (p = 0.66) achieved MRD negativity prior to transplant. Among patients with available post-transplant MRD data, 50% (6/12) of older patients and 75% (45/60) of younger patients achieved MRD negativity (p = 0.10). Median time to neutrophil engraftment was 12 days in both age groups (range 10–15 vs 10–13; p = 0.06), and median time to platelet engraftment was 14 days (10–18) in the older group vs 13 days (9–20) in the younger group (p = 0.09) (Supplementary Table 1).

Median follow-up was 19.1 months (range 3.6-47.4), 15.0 months in the older group and 19.9 months in the younger group. Median PFS was 36.5 months in the older group and not reached in the younger group, while OS was not reached in either cohort. One- and two-year PFS rates were 91% and 78% in the older group, compared to 93% and 89% in the younger group (p = 0.41). One- and two-year OS rates were 100% and 92% in older patients versus 98% and 93% in younger patients. In IPW-adjusted analyses, there was no significant difference in PFS (hazard ratio [95% CI] 1.30 [0.36–4.67]; 0.69) or OS (0.49 [0.02–14.29]; p = 0.68) between the two age groups.

No significant predictors of PFS or OS were identified through univariate analyses (Supplementary Tables 2 and 3, respectively). There was a trend toward improved OS among patients who received ≥4 cycles of induction therapy (0.20 [0.03–1.21]; p = 0.08) and those who received post-transplant maintenance therapy (0.17 [0.03–1.05]; p = 0.057).

Our findings demonstrate that older adults (≥70 years) with newly diagnosed MM treated with D-VRD induction followed by autoHCT achieve similar response rates, short-term PFS and OS to those of younger patients, with no treatment-related mortality. These results are consistent with those from the GRIFFIN [2] and PERSEUS [1] trials, which evaluated D-VRD in transplant-eligible patients, but excluded those over age 70. In GRIFFIN, patients who received D-VRD had post-transplant ≥CR and ≥VGPR rates of 27% and 87%, respectively, which deepened to 63% and 80% at last follow-up. MRD negativity was achieved in 21% post-induction and 51% post-transplant. Similarly, in PERSEUS, the D-VRD arm demonstrated a ≥CR rate of 88% and post-transplant MRD negativity in 75% of patients. However, the median ages in GRIFFIN and PERSEUS were 59 and 61 years, respectively. Moreover, high-risk cytogenetics were present in only 15% of patients in GRIFFIN and 21% in PERSEUS, limiting the generalizability of these findings for higher-risk or older populations. In contrast, our real-world cohort included patients up to age 78, with a substantially higher proportion of patients with high-risk cytogenetic abnormalities. Even with these adverse characteristics, patients in this report achieved similar deep responses and MRD negativity rates, reinforcing the feasibility of D-VRD induction followed by autoHCT in appropriately selected older adults. Median PFS in our cohort was shorter than in GRIFFIN or PERSEUS, likely reflecting the higher proportion of high-risk cytogenetic abnormalities, broader clinical and treatment variability in real-world populations, and a shorter follow-up.

Prior studies conducted in the pre-quadruplet era demonstrated encouraging outcomes with autoHCT in older adults. For example, a retrospective analysis from Stanford evaluated patients ≥70 years old, who were referred for autoHCT between 2010 and 2019. They showed a superior PFS (41 vs 33 months, p = 0.03), no early transplant-related mortality, and similar OS as matched non-transplant controls [5]. However, only about half of these patients received an immunomodulatory drug + proteasome inhibitor-based induction, and anti-CD38 antibodies were not widely used at that time. Similarly, an Emory University study of patients aged ≥75 years, who underwent an autoHCT between 2006 and 2016 demonstrated significantly longer median PFS (50 vs 30 months) and OS (80 vs 40 months) compared to non-transplant controls, again with no transplant-related mortality [6]. Single-center experiences from Mayo Clinic [7] and MD Anderson [8] have also shown the safety and feasibility of autoHCT in selected patients aged ≥75 years, with low NRM and encouraging outcomes.

Moreover, a CIBMTR registry study that included patients undergoing autoHCT between 2013 and 2017 showed that those aged ≥70 years had similar relapse rates, PFS, and OS as patients aged 60–69 years, underscoring the value of autoHCT across age groups in contemporary settings [9]. Our results compare favorably to these prior studies, highlighting the safety and feasibility of upfront autoHCT after quadruplet induction in selected older adults.

Importantly, no randomized trial has directly compared transplant versus non-transplant approaches specifically in older adults, either in the modern treatment setting or historically. Given the underrepresentation of older adults in clinical trials and growing evidence that age disparities in oncology studies are pervasive and worsening [10], our study provides important real-world evidence supporting autoHCT as a safe and effective consolidation strategy in this population, even in patients up to age 78.

Our study has the characteristic limitations of a retrospective analysis, including potential selection bias and missing data, as only patients referred for and deemed eligible for transplant were included, likely representing a healthier subset of older adults. The sample size of patients aged ≥70 years was relatively small, limiting statistical power and the precision of effect estimates, and the study may be underpowered to detect subtle differences between the two age groups. Variability in induction and conditioning regimens between the two groups, along with the lack of availability of key measures such as frailty, may introduce residual confounding despite inverse probability weighting. Additionally, key treatment variables, such as bortezomib dose and schedule and standardized frailty assessments were not routinely collected and, therefore, could not be analyzed. Furthermore, post-transplant MRD data were unavailable for many patients, and the follow-up was relatively short. Finally, in the absence of a non-transplant comparator arm, our study cannot evaluate the relative benefit of autoHCT versus non-transplant approaches in older patients in the modern treatment era.

In conclusion, this study showed that in selected older adults D-VRD induction followed by autoHCT is safe, feasible, and is associated with excellent outcomes with available follow up.

## Supplementary information

Supplement

## Data Availability

The data that support the findings of this study are available on request to the corresponding author.
