# Supplementary material for: D-VRD induction and autologous transplant in patients ≥70 years
Source: Blood Cancer J. 2026 Jun 5;16(1):91. doi: 10.1038/s41408-026-01522-x (PMC13241521; doi:10.1038/s41408-026-01522-x)
Supplement: Supplementary file 1 — Supplement [file 41408_2026_1522_MOESM1_ESM.docx]

**Supplementary Table 1. Summary of Engraftment Kinetics**

|  |  | **Age Category** | | |
| --- | --- | --- | --- | --- |
| **Engraftment** | **All patients**  **(N=125)** | **<70 years**  **(N=98)** | **≥70 years**  **(N=27)** | **p-value^a^** |
| **Days to ANC > 500** |  |  |  |  |
| Median (range) | 12 (10 – 15) | 12 (10 – 13) | 12 (10 – 15) | 0.06 |
| **Days to platelet counts > 20k** |  |  |  |  |
| Median (range) | 13 (9 – 20) | 13 (9 – 20) | 14 (10 – 18) | 0.09 |

^a^ Wilcoxon rank sum test

**Abbreviations**: ANC =absolute neutrophil count

**Supplementary Table 2. Summary of univariate assessments for progression-free survival**

|  | **Progression-Free Survival** | |
| --- | --- | --- |
|  | **Hazard Ratio (95% CI)** | ***p*-value** |
| **Age at autoHCT** |  |  |
| ≥70 years vs <70 years | 1.54 (0.55 **–** 4.34) | 0.41 |
| **Gender** |  |  |
| Female vs Male | 0.78 (0.29 – 2.09) | 0.62 |
| **Race** |  |  |
| Non-Black vs Black | 1.55 (0.35 – 6.82) | 0.56 |
| **R-ISS** |  |  |
| II vs I  III vs I | 1.07 (0.27 – 4.31)  3.80 (0.90 – 16.12) | 0.92  0.07 |
| **R2-ISS** |  |  |
| II vs I  III vs I  IV vs I | 0.41 (0.04 – 3.96)  1.01 (0.25 – 4.08)  3.86 (0.77 – 19.40) | 0.44  0.99  0.10 |
| **Number of Induction cycles** |  |  |
| ≥ 4 vs < 4 | 0.39 (0.13 – 1.20) | 0.10 |
| **Duration of induction** |  |  |
| ≥ 126 days vs < 126 days | 0.77 (0.30 – 2.02) | 0.60 |
| **Most common lenalidomide dose** |  |  |
| ≥ 20 mg vs < 20 mg | 0.38 (0.15 – 0.97) | 0.043 |
| **Maximum lenalidomide dose** |  |  |
| ≥ 20 mg vs < 20 mg | 0.62 (0.22 – 1.76) | 0.37 |
| **Cytogenic risk** |  |  |
| High vs Standard | 0.93 (0.37 – 2.37) | 0.88 |
| **ECOG performance status** |  |  |
| 1 vs 0 | 0.42 (0.14 – 1.27) | 0.12 |
| **HCT-Cl** |  |  |
| > 3 vs ≤ 3 | 0.53 (0.17 – 1.62) | 0.27 |
| **Response prior to autoHCT** |  |  |
| VGPR vs CR  PR vs CR | 0.96 (0.30 – 3.05)  0.58 (0.13 – 2.61) | 0.95  0.48 |
| **MRD prior to auto-HCT** |  |  |
| Positive vs Negative | 1.94 (0.68 – 5.55) | 0.22 |
| **MRD negative ≥ VGPR prior to autoHCT** |  |  |
| Yes vs No | 0.66 (0.23 – 1.87) | 0.43 |
| **Conditioning Regimen** |  |  |
| Busulfan + Melphalan vs Melphalan  Melphalan hydrochloride vs Melphalan | 0.87 (0.28 – 2.70)  1.20 (0.27 – 5.42) | 0.81  0.81 |
| **Best response^a^** |  |  |
| CR vs non-CR | 0.77 (0.29 – 2.09) | 0.61 |
| **Best MRD status post autoHCT^a^** |  |  |
| Positive vs Negative | 0.95 (0.25 – 3.53) | 0.94 |
| **Maintenance^a^** |  |  |
| Yes vs No | 2.03 (0.49 – 8.37) | 0.33 |

^a^ Included in the model as a time-dependent covariate.

**Abbreviations**: autoHCT = autologous hematopoietic cell transplantation; CR = complete response; HCT-CI = Hematopoietic Cell Transplantation Comorbidity Index; MRD = measurable residual disease; PR = partial response; R-ISS = Revised International Staging System; VGPR = very good partial response.

**Supplementary Table 3. Summary of univariate assessments for overall survival**

|  | **Overall Survival** | |
| --- | --- | --- |
|  | **Hazard Ratio (95% CI)** | ***p*-value** |
| **Age at autoHCT** |  |  |
| ≥70 years vs <70 years | 1.11 (0.12 – 9.97) | 0.92 |
| **Gender** |  |  |
| Female vs Male | 2.58 (0.43 – 15.44) | 0.30 |
| **Race** |  |  |
| Non-Black vs Black | 2.16 (0.09 – 51.59) | 0.64 |
| **R-ISS** |  |  |
| II vs I  III vs I | 1.63 (0.03 – 84.05)  14.87 (0.33 – 664.4) | 0.81  0.16 |
| **R2-ISS** |  |  |
| II vs I  III vs I  IV vs I | 1.41 (0.01 – 362.5)  1.66 (0.02 – 154.8)  11.99 (0.13 – 1116) | 0.90  0.83  0.28 |
| **Number of Induction cycles** |  |  |
| ≥ 4 vs < 4 | 0.20 (0.03 – 1.21) | 0.08 |
| **Duration of induction** |  |  |
| ≥ 126 days vs < 126 days | 0.30 (0.03 – 2.67) | 0.28 |
| **Most common lenalidomide dose** |  |  |
| ≥ 20 mg vs < 20 mg | 0.56 (0.09 – 3.36) | 0.52 |
| **Maximum lenalidomide dose** |  |  |
| ≥ 20 mg vs < 20 mg | 0.86 (0.10 – 7.72) | 0.89 |
| **Cytogenic risk** |  |  |
| High vs Standard | 5.12 (0.57 – 45.92) | 0.14 |
| **ECOG performance status** |  |  |
| 1 vs 0 | 0.12 (0.00 – 4.90) | 0.26 |
| **HCT-CI** |  |  |
| > 3 vs ≤ 3 | 2.91 (0.49 – 17.43) | 0.24 |
| **Response prior to autoHCT** |  |  |
| VGPR vs CR  PR vs CR | 0.27 (0.04 – 1.73)  0.12 (0.00 – 3.45) | 0.17  0.21 |
| **MRD prior to auto-HCT** |  |  |
| Positive vs Negative | 0.27 (0.03 – 2.58) | 0.25 |
| **MRD negative ≥ VGPR prior to autoHCT** |  |  |
| Yes vs No | 5.00 (0.52 – 48.31) | 0.16 |
| **Conditioning Regimen** |  |  |
| Busulfan + Melphalan vs Melphalan  Melphalan hydrochloride vs Melphalan | 1.90 (0.30 – 12.12)  0.73 (0.02 – 21.99) | 0.50  0.86 |
| **Best response^a^** |  |  |
| CR vs non-CR | 1.75 (0.19 – 16.26) | 0.62 |
| **Best MRD status post autoHCT^a^** |  |  |
| Positive vs Negative | 1.23 (0.08 – 19.68) | 0.88 |
| **Maintenance^a^** |  |  |
| Yes vs No | 0.17 (0.03 – 1.05) | 0.057 |

^a^ Included in the model as a time-dependent covariate.

**Abbreviations**: autoHCT = autologous hematopoietic cell transplantation; CR = complete response; HCT-CI = Hematopoietic Cell Transplantation Comorbidity Index; MRD = measurable residual disease; PR = partial response; R-ISS = Revised International Staging System; VGPR = very good partial response.
